# Case report: Treatment of tachycardia-induced cardiogenic shock with permanent His bundle pacing and atrioventricular node ablation

**DOI:** 10.3389/fcvm.2022.992675

**Published:** 2022-11-14

**Authors:** Tadej Žlahtič, Miša Fister, Peter Radšel, Marko Noč, Matjaž Šinkovec, David Žižek

**Affiliations:** ^1^Department of Cardiology, University Medical Centre Ljubljana, Ljubljana, Slovenia; ^2^Department of Intensive Internal Medicine, University Medical Centre Ljubljana, Ljubljana, Slovenia

**Keywords:** His bundle pacing, cardiogenic shock, mechanical circulatory support, cardiac resynchronization therapy, tachycardia-induced cardiomyopathy, AV node ablation

## Abstract

Tachycardia-induced cardiomyopathy (T-CMP) related to supraventricular arrhythmia is a rare and often unrecognized cause of refractory cardiogenic shock. When rhythm control interventions are ineffective or no longer pursued, atrioventricular node ablation (AVNA) with pacemaker implantation is indicated. Conduction system pacing provides normal synchronous activation of the ventricles after AVNA. However, there is a lack of data on pace and ablate strategy in hemodynamically unstable patients. We report on 2 patients with T-CMP presenting with refractory cardiogenic shock who were successfully treated with His bundle pacing in conjunction with AVNA.

## Introduction

Tachycardia-induced cardiomyopathy (T-CMP) is defined as the presence of reversible left ventricular (LV) dysfunction due to persistent rapid ventricular rate, regardless of tachycardia etiology (1). The common causes of T-CMP are supraventricular arrhythmias, namely atrial fibrillation (AF), atrial flutter, and atrial tachycardia (1, 2). Most of the patients present with heart failure (HF) symptoms and palpitations, while cardiogenic shock and cardiac arrest remain relatively rare (1). Treatment of T-CMP consists of suppression of ventricular rate with antiarrhythmic drugs (AADs), arrhythmia elimination with radiofrequency ablation (RFA) or electrical cardioversion (EC), and atrioventricular node ablation (AVNA) with pacemaker implantation when rhythm control interventions are ineffective or no longer pursued (2). Recently, conduction system pacing was introduced into clinical practice which, in contrast to standard right ventricular (RV), provides normal synchronous activation and preserves LV function in HF patients (3). However, evidence for the use of “ablate and pace” strategy with HBP in the T-CMP presenting with cardiogenic shock are scarce.

We report two patients with T-CMP who were admitted to the intensive care unit (ICU) due to cardiogenic shock and were successfully treated with HBP and AVNA.

## Case report

### Case 1

**Figure 1:**
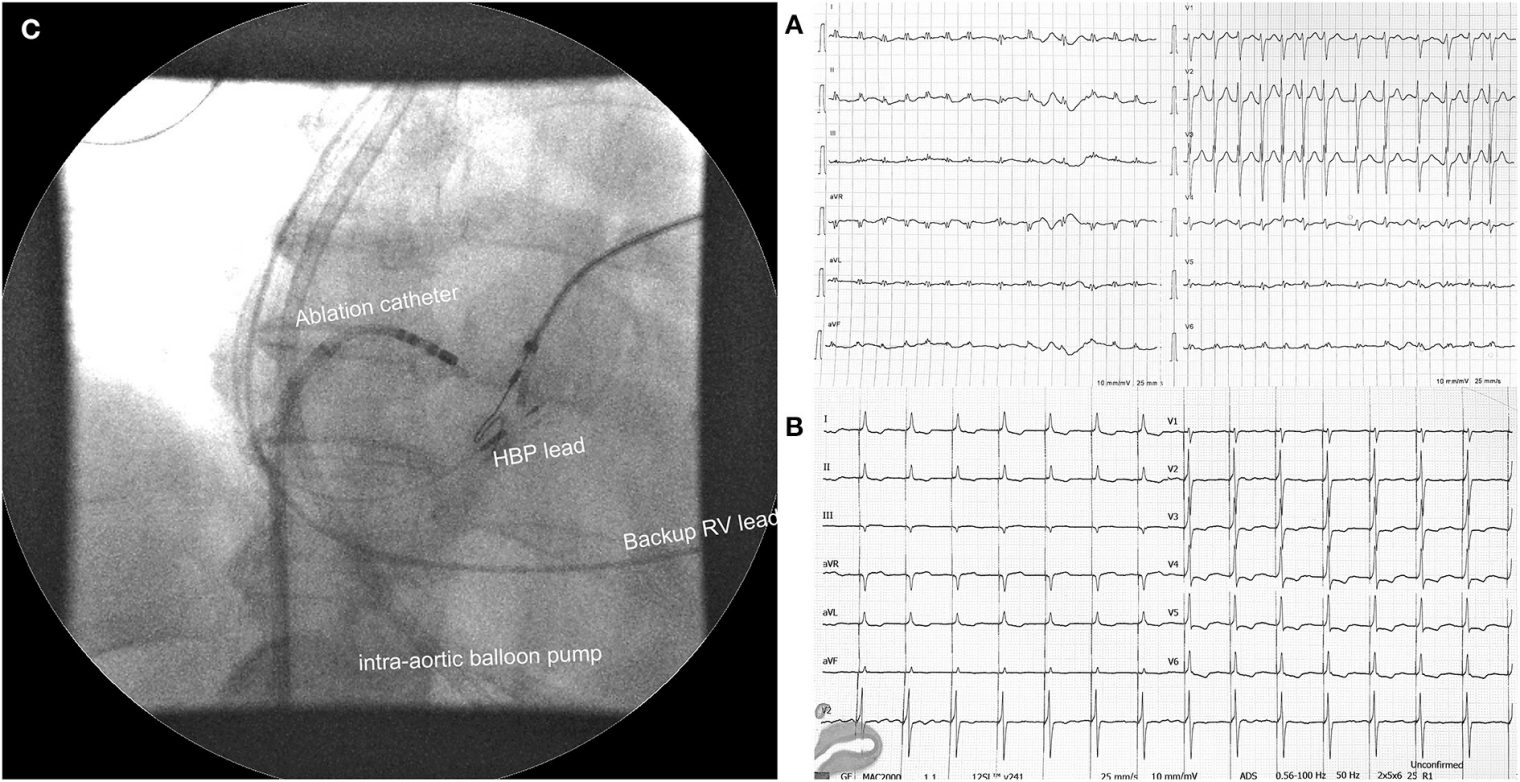
**(A)** Electrocardiogram at admission with visible atrial fibrillation with a ventricular rate of 150 bpm. **(B)** Electrocardiogram after atrioventricular node ablation and His bundle pacing. **(C)** Position of pacing leads and ablation catheter during fluoroscopy with visible intra-aortic balloon pump in the background. HBP, His bundle pacing; RV, right ventricle.

A 65-year-old woman with a history of AF, diabetes type II, and ischemic CMP was admitted due to progressive dyspnea and peripheral oedema. As she was not attending regular outpatient clinic follow-ups, the level of heart rate control or the duration of AF was not well-established. On examination at the emergency department, she was hypotensive (93/56 mmHg) with signs of cardiogenic shock. A 12-lead ECG revealed AF with a ventricular rate of around 150 bpm (Figure 1). Bedsides, echocardiography showed severely dilated LV with severely reduced EF and dilated right ventricle (RV) with reduced systolic function. The left atrium was severely dilated (Table 1). Laboratory findings showed metabolic acidosis (pH 7.32), increased lactate levels (11,4 mmol/L), acute kidney injury [creatinine levels of 164 μmol/l, glomerular filtration rate (GFR) 28 mL/min/1,73m^2^], severely elevated transaminases with international normalized ratio (INR) of 7, negative troponin, elevated NT-proBNP (7,024 pg/mL), and normal inflammatory markers. Initial supportive intravenous therapy did not result in clinical improvement. Invasive mechanical ventilation (MV) was initiated together with inhaled nitric oxide due to concomitant RV failure. Landiolol infusion resulted in a moderate heart rate decline from 170 to 140 bpm but shock persisted. Coronary angiography did not reveal obstructive coronary lesions. Intra-aortic balloon pump (IABP) was inserted but resulted in low augmented pressure due to tachycardia. Laboratory tests showed normal thyroid function. Bilateral stellate ganglion blockade did not result in a significant heart rate decrease. After 1 week of hospitalization and several unsuccessful synchronized EC, the “ablate and pace” strategy was attempted. Non-selective HBP was achieved with a stable pacing threshold of 2.25 V at 1 ms (Figure 1). There were no procedure-related complications. Hours after the procedure there was a significant improvement in LV function. Twenty-four h after the procedure her condition improved and IABP could be removed. We started with low dose HF therapy. Her condition further improved, and she was weaned from MV on day 17. Her blood pressure normalized, and LV function further improved. She was discharged from the hospital on day 43. At 1-year follow-up, her condition was stable (Table 1).

**Table 1:** Summary of clinical presentations, cardiac function, and pacing parameters.

	**Case 1**	**Case 2**
Clinical presentation	Cardiogenic shock with multiorgan failure needing mechanical circulatory support, on intra-aortic balloon pump, pulmonary oedema needing mechanical ventilation.	Cardiogenic shock with multiorgan failure on VA-ECMO, pulmonary oedema needing mechanical ventilation, thyrotoxicosis.
History	Ischemic cardiomyopathy (LVEF 55%), permanent atrial fibrillation	None
Medications	Warfarin, bisoprolol 7.5 mg q.d., furosemide 60 mg q.d., rosuvastatin 40 mg q.d.. Perindopril, spironolactone, and metildigoxin were canceled 1 week before admission due to worsening kidney function, hypotension, and high digoxin levels	No regular medication
Admission	EDV 140 ml LVEF 10% LVOT VTI 6 cm TAPSE 0.9 cm LAVI 57 ml/m2	EDV 170 ml LVEF 10% LVOT VTI 4 cm TAPSE 0.8 cm LAVI 52 ml/m2
After the procedure	EDV 140 ml LVEF 25% LVOT VTI 13 cm TAPSE 1.1 cm HBP threshold 2.25V@1 ms, impedance 418 Ohm Fluoroscopic time: 20 min Procedure duration: 90 min	EDV 170 ml LVEF 30% LVOT VTI 13 cm TAPSE 1.4 cm HBP threshold 0.75V@1 ms, impedance 510 Ohm Fluoroscopic time: 4.5 min Procedure duration: 50 min
At discharge	EDV 120 ml LVEF 39% LVOT VTI 15 cm TAPSE 1.3 cm HBP threshold 2.75V@1 ms	EDV 150 ml LVEF 45% LVOT VTI 20 cm TAPSE 2.4 cm HBP threshold 1.25V@1 ms
1 year follow up	EDV 116 ml LVEF 46% LVOT VTI 15 cm TAPSE 1.4 cm HBP threshold 3V@1 ms	EDV 140 ml LVEF 51% LVOT VTI 15 cm TAPSE 2.3 cm HBP threshold 1.5V@1 ms

VA-ECMO, veno-arterial extracorporeal membrane oxygenation; LVEF, left ventricular ejection fraction; EDV, end diastolic volume; LAVI, left atrial volume index; LVOT VTI, left ventricular outflow tract velocity time integral; TAPSE, tricuspid annular plane systolic excursion; HBP, His bundle pacing.

### Case 2

**Figure 2:**
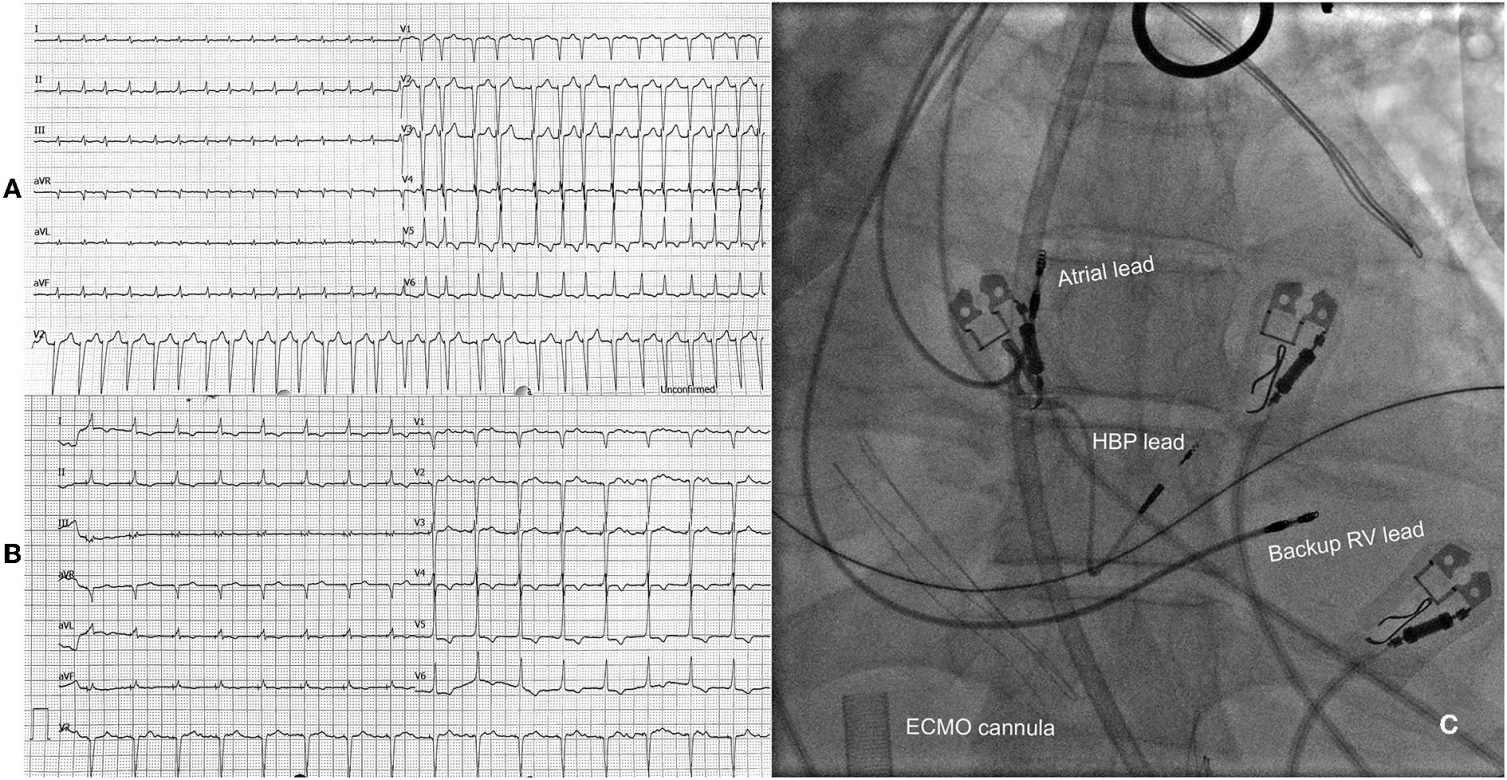
**(A)** Electrocardiogram at admission with visible atrial fibrillation with a ventricular rate of 175 bpm. **(B)** Electrocardiogram after atrioventricular node ablation and His bundle pacing. **(C)** Position of pacing leads during fluoroscopy with visible ECMO cannula in the background. HBP, His bundle pacing; RV, right ventricle; ECMO, extracorporeal membrane oxygenation.

A 51-year-old, previously healthy male, visited the emergency department due to progressive weakness and palpitations. On examination, he was hypotensive (94/73 mmHg) with signs of cardiogenic shock. The electrocardiogram showed AF with a rapid ventricular rate of 175 bpm (Figure 2). Laboratory results showed elevated lactate levels (3.6 mmol/L) with metabolic acidosis (pH 7.29), severely elevated transaminases, acute kidney injury (creatinine 226 μmol/l, GFR 28 mL/min/1,73m^2^) with moderate hyperkaliemia (6 mmol/l). Within hours, the patient's status further deteriorated. Invasive MV was initiated, and a high dose of norepinephrine was needed. Transesophageal echo revealed dilated LV, enlarged atria, and severely decreased ventricular contractility (Table 1). No thrombi were found in the left atrium appendage. A coronary angiogram revealed non-obstructive coronary artery disease. Several synchronized EC attempts and intravenous AADs were unsuccessful in eliminating arrhythmia or markedly decreasing the ventricular rate. Due to refractory cardiogenic shock, peripheral percutaneous veno-arterial extracorporeal membrane oxygenation (VA-ECMO) was inserted. Further laboratory analysis confirmed elevated levels of thyroxin. Intravenous steroids and thiamazole were initiated. However, multi-organ failure worsened rapidly and 4,5 L/min of ECMO flow was insufficient. Thus, we proceeded with an urgent “ablate and pace” strategy. His bundle pacing lead was inserted and selective HBP was achieved with a stable threshold of 0.75V at 1 ms. A return to sinus rhythm and significant improvement of cardiac function was expected, atrial and RV backup leads were also inserted and connected to the atrio-biventricular device (Figure 2). Within an hour after the procedure, the pulsatile flow was noted. The norepinephrine dose was lowered, and cardiac function improved significantly after 24 h. On day 5, VA-ECMO could be removed, and low dose HF therapy was initiated. Further ICU stay was prolonged due to bacterial ventilator associated pneumonia, gastric perforation, fungal infection, and critical illness myopathy with long ventilation weaning. The patient was discharged from hospital on day 47 with significantly improved cardiac function and sinus rhythm. At the 1-year follow-up, his condition was stable (Table 1).

### His bundle pacing and atrioventricular node ablation procedure

In both presented cases device implantation was performed first followed by AVNA during the same procedure as previously described (4). We used SelectSecure 3,830 (Medtronic, Minneapolis, USA) active fixation leads and dedicated delivery sheaths. His bundle area mapping was performed in a unipolar setting with LAB system Pro (BARD/Boston Scientific, Lowell, USA) electrophysiological system. Atrioventricular node ablation was done with irrigated Flexability™ (Abbott, USA) or Celsius^®^ Thermocool^®^ (Biosense Webster, USA) tip ablation catheter in a temperature-controlled mode (40 W, up to 60 s). The lower rate of the pacing device was initially set to 80 bpm and programmed to 70 bpm at follow-up.

## Discussion

With our case series, we were able to show that HBP in conjunction with AVNA could present a feasible and safe treatment option even in the T-CMP presenting with cardiogenic shock.

In a recent retrospective analysis, Hékimian et al. showed the feasibility of the “ablate and pace” strategy with temporary septal RV and later conversion to BiV pacing in patients with T-CMP requiring mechanical circulatory support (5). Nonetheless, the “ablate and pace” strategy is generally utilized in hemodynamically stable symptomatic patients with supraventricular tachycardia refractory to pharmacological therapy and RFA (2). However, several studies reported neutral findings regarding HF progression and survival, implying that the beneficial effects of rate control after AVNA could be hampered by non-physiologic dyssynchronous RV pacing (6). While biventricular pacing in conjunction with AVNA has shown better results compared to RV pacing, the benefit was much less distinct in patients with narrow QRS or normal left ventricular function (3, 6). By stimulating the native conduction system through a bundle of His, normal synchronous activation of the ventricles can be obtained. Therefore, it could represent an alternative to BiV pacing in patients with an expected high percentage of pacing with concomitant severe left ventricular dysfunction and narrow QRS (3, 7). Recent randomized trials further confirmed that HBP could deliver better improvement of EF compared to BiV in patients undergoing AVNA (8). However, there are some limitations associated with HBP, e.g.: higher capture thresholds, need for RV back-up lead, lower success rates, etc (4). Thus, left bundle branch area pacing could present an even better physiological pacing option to overcome these limitations, especially in the setting of AVNA (9).

An alternative approach could have adopted rhythm control with pulmonary vein isolation (PVI). Catheter ablation of AF has become a well-established procedure in HF patients as sinus rhythm restoration significantly lowers the rate of death or hospitalization for worsening heart failure compared with medical therapy alone (10, 11). However, there is still insufficient data on ablation in case of hemodynamic instability due to AF. Mantini et al. (11) reported successful ablation of atrial arrhythmias in five patients with cardiogenic shock on mechanical circulatory support. Although there were no complications reported, there were concerns about the safety of the procedure in critically ill patients with rapidly progressing cardiogenic shock (11). Ablation procedures in persistent AF are no more than 20–60% successful in maintaining the sinus rhythm. Furthermore, several patient characteristics play an important role in AF ablation success rates, for example, the need for high direct current energies for the restoration of sinus rhythm in cardioversion prior to ablation, left atrial size, AF duration, patient age, renal dysfunction, and substrate visualized on magnetic resonance imaging (10, 11). Therefore, it is conceivable to assume that the AF ablation strategy with PVI in our presented cases would not yield a significant probability of acute sinus rhythm restoration, especially in the setting of cardiogenic shock. Similar observations were noted by Hékimian et al., where only 1 ablation procedure was performed in 35 patients presenting with T-CMP and cardiogenic shock (5).

In conclusion, in the T-CMP presenting with cardiogenic shock “ablate and pace” strategy with HBP could present a feasible and safe treatment option for arrhythmia reduction. Further clinical studies are warranted to address the best strategy for addressing the severest forms of T-CMP.

## Data availability statement

The original contributions presented in the study are included in the article/supplementary material, further inquiries can be directed to the corresponding author/s.

## Ethics statement

Written informed consent was obtained from the individual(s) for the publication of any potentially identifiable images or data included in this article.

## Author contributions

TŽ and DŽ have written majority of the manuscript. MF and PR contributed to conception and wrote sections of the manuscript. MN and MŠ have equally contributed to the manuscript revision. All authors contributed to manuscript revision, read, and approved the submitted version.

## Conflict of interest

The authors declare that the research was conducted in the absence of any commercial or financial relationships that could be construed as a potential conflict of interest.

## Publisher's note

All claims expressed in this article are solely those of the authors and do not necessarily represent those of their affiliated organizations, or those of the publisher, the editors and the reviewers. Any product that may be evaluated in this article, or claim that may be made by its manufacturer, is not guaranteed or endorsed by the publisher.

## References

[1] Huizar JF Ellenbogen KA Tan AY Kaszala K. Arrhythmia-induced cardiomyopathy. J Am Coll Cardiol. (2019) 73:2328–44. 10.1016/j.jacc.2019.02.045 31072578 PMC6538508

[2] Brugada J Katritsis DG Arbelo E Arribas F Bax JJ Blomström-Lundqvist C. 2019 esc guidelines for the management of patients with supraventricular tachycardiathe task force for the management of patients with supraventricular tachycardia of the European society of cardiology (Esc): developed in collaboration with the association for European paediatric and congenital cardiology (Aepc). Eur Heart J. (2019) 41:655–720. 10.1093/eurheartj/ehz467 31504425

[3] Huang W Su L Wu S Xu L Xiao F Zhou X. Benefits of permanent his bundle pacing combined with atrioventricular node ablation in atrial fibrillation patients with heart failure with both preserved and reduced left ventricular ejection fraction. J Am Heart Assoc. (2017) 6:e005309. 10.1161/JAHA.116.005309 28365568 PMC5533020

[4] Vijayaraman P Chung MK Dandamudi G Upadhyay GA Krishnan K Crossley G. His bundle pacing. J Am Coll Cardiol. (2018) 72:927–47. 10.1016/j.jacc.2018.06.017 30115232

[5] Hékimian G Paulo N Waintraub X Bréchot N Schmidt M Lebreton G. Arrhythmia-induced cardiomyopathy: a potentially reversible cause of refractory cardiogenic shock requiring venoarterial extracorporeal membrane oxygenation. Heart Rhythm. (2021) 18:1106–12. 10.1016/j.hrthm.2021.03.014 33722763

[6] Stavrakis S Garabelli P Reynolds DW. Cardiac resynchronization therapy after atrioventricular junction ablation for symptomatic atrial fibrillation: a meta-analysis. EP Europace. (2012) 14:1490–7. 10.1093/europace/eus193 22696519

[7] Žižek D Antolič B Mežnar AZ Zavrl-Džananović D Jan M Štublar J. Biventricular versus his bundle pacing after atrioventricular node ablation in heart failure patients with narrow qrs. Acta Cardiol. (2021) 2021:1–9. 10.1080/00015385.2021.1903196 34078244

[8] Huang W Wang S Su L Fu G Su Y Chen K. His-bundle pacing vs biventricular pacing following atrioventricular nodal ablation in patients with atrial fibrillation and reduced ejection fraction: a multicenter, randomized, crossover study—the alternative-af trial. Heart Rhythm. (2022) 14:S1547-5271(22)02172-5. 10.1016/j.hrthm.2022.07.009 35843465

[9] Zhang S Zhou X Gold MR. Left bundle branch pacing. J Am Coll Cardiol. (2019) 74:3039–49. 10.1016/j.jacc.2019.10.039 31865972

[10] Hindricks G Potpara T Dagres N Arbelo E Bax JJ Blomström-Lundqvist C. 2020 esc guidelines for the diagnosis and management of atrial fibrillation developed in collaboration with the European association of cardio-thoracic surgery (Eacts): the task force for the diagnosis and management of atrial fibrillation of the European society of cardiology (Esc) developed with the special contribution of the European heart rhythm association (Ehra) of the esc. Eur Heart J. (2020) 42:373–498. 10.1093/eurheartj/ehaa612 32860505

[11] Mantini N Zipse M Tompkins C Varosy PD Sauer WH Nguyen DT. Ablation of atrial arrhythmias in patients with cardiogenic shock on mechanical circulatory support. HeartRhythm Case Rep. (2018) 5:115–9. 10.1016/j.hrcr.2018.11.008 30891405 PMC6404096

